# The Anatomy of Facial Muscles Revisited: High-Resolution Magnetic Resonance Imaging and Computed Tomography Studies on Body Donors

**DOI:** 10.3390/jimaging12090459

**Published:** 2026-09-20

**Authors:** Heiko Stark, Kenny Jandausch, Angelina Roth, Uta Biedermann, Martin Krämer, Jürgen R. Reichenbach, Rene Aschenbach, Gerd Fabian Volk, Martin S. Fischer, Orlando Guntinas-Lichius

**Affiliations:** 1 Institute of Zoology and Evolutionary Research with Phyletic Museum, Friedrich-Schiller-University Jena, 07737 Jena, Germany; martin.s.fischer.jena@gmail.com; 2 Department of Otorhinolaryngology, Jena University Hospital, 07740 Jena, Germanyfabian.volk@med.uni-jena.de (G.F.V.); orlando.guntinas@med.uni-jena.de (O.G.-L.); 3 Institute of Anatomy, Jena University Hospital, 07740 Jena, Germany; uta.biedermann@med.uni-jena.de; 4 Institute of Diagnostic and Interventional Radiology, Jena University Hospital, 07740 Jena, Germany; martin.kraemer@med.uni-jena.de (M.K.); rene.aschenbach@med.uni-jena.de (R.A.); 5 Medical Physics Group, Institute of Diagnostic and Interventional Radiology, Jena University Hospital, 07740 Jena, Germany; juergen.reichenbach@med.uni-jena.de; 6 Facial-Nerve-Center, Jena University Hospital, 07740 Jena, Germany; 7 Center for Rare Diseases, Jena University Hospital, 07740 Jena, Germany

**Keywords:** face, mimic, facial muscles, facial nerve, imaging, magnetic resonance imaging, computed tomography, topography, anatomy

## Abstract

The facial muscles are crucial for nonverbal communication, physiological functions, and articulation. Unlike skeletal or masticatory muscles, they lack fascia, attach directly to the skin and function without joint-mediated movements or visual feedback. The aim of this study was to reconstruct the facial musculature using high-resolution computerised tomography (CT) and 3 Tesla magnetic resonance imaging (MRI) on 14 body donors, yielding segmented 3D datasets of the facial muscles, as well as bony and soft-tissue head structures. We hypothesise that the facial musculature comprises functional subunits with variable geometries that extend beyond classical anatomical definitions, with attachment points and three-dimensional positioning that differ from conventional descriptions. The most significant finding was that MRI could not resolve individual facial muscles as depicted in anatomical atlases. Instead, the musculature appeared as a single continuous sheet of muscle, embedded in fatty tissue and not attached to the bone. Whether the facial musculature is in fact continuous or consists of several muscles connected by connective tissue remains unclear. The difficulty of segmentation varied depending on the region; the zygomatic muscle was the easiest to separate, whilst the orbicularis oculi, frontalis and platysma muscles proved particularly difficult due to their thin, flat morphology.

## 1. Introduction

The facial muscles are crucial to our nonverbal communication. Without facial muscles, a person is socially isolated (nonverbal feedback), physiologically impaired (swallowing and eye problems), and linguistically restricted (articulation). Preventing or mitigating problems requires understanding the detailed structure and function of the facial muscles. The facial muscles form a complex, interconnected network. They lack fascia and are connected to the periosteum of the facial skull (with the exception of the *M. frontalis* and *M. orbicularis oris*) and directly to the skin [1,2]. Because muscles can contract, they influence facial geometry. Distinctive features compared to the somatic musculature, e.g., of the extremities, include the absence of joints and the lack of visual feedback regarding movement trajectories. The muscles are controlled by the central nervous system via a voluntary motor system and a spontaneous-affective system. The facial muscle network is traditionally divided into approximately 20 anatomically defined muscle pairs. These muscles are usually divided functionally into ‘facial muscle units’ around the facial orifices (eyes, nose, mouth). Due to the typical topographical illustrations of the musculature in anatomical atlases, muscular movements are viewed in two dimensions [3]. Neither aspect adequately reflects the function. Facial movements or emotional expressions do not correspond to the classical muscle structures defined by gross anatomical dissections and are three-dimensional across the entire face [4].

These movements differ fundamentally from those of the somatic musculature and also from those of the masticatory muscles. Whilst the masticatory muscles are optimised for force transmission and load-bearing, the facial muscles serve as a system for the precise deformation of soft tissues. A key biomechanical difference lies in the type of muscle attachment, whether to relatively fixed points such as bone or to the skin or other muscles [5,6]. When a facial muscle contracts, it does not simply pull at a single point but transmits its tension via a network across an entire area of skin [7]. As the facial muscles attach to the soft skin, every contraction generates a directed force that moves and deforms the tissue. This becomes more interesting at the modiolus, the mechanical ‘junction’ at the angle of the mouth where up to nine muscle vectors converge. In a real smile (Duchenne smile), two main vectors (the zygomatic and orbital vectors) interact, causing a complex displacement of tissue [8,9].

Considering the clinical context and biomechanics, the aim of this study was to reconstruct facial muscles using body donors and applicable methods, with a future application in healthy volunteers and patients. This includes computed tomography (CT) and magnetic resonance imaging (MRI), as both methods are established in everyday clinical practice for 3D imaging [10]. These imaging techniques allow the musculature and neighbouring structures to be visualised and digitised [11,12]. It is well known that the resolution in some regions of the body is insufficient to enable precise differentiation between different anatomically defined muscles [13,14]. Combining the imaging of hard and soft tissues with specific MR protocols can improve tissue differentiation [11]. However, this must be balanced against the available scanning time and the associated radiation and heating exposures, resulting in a trade-off between the two approaches. The data obtained in this way can then serve as the basis for models to visualise and simulate facial musculature [15]. The question of whether CT and MRI are sufficient to identify facial muscles within the defined topography, as has been the practice to date, is the focus of this study.

In short, our hypothesis is that the facial musculature comprises functional subunits with variable geometries beyond the traditional definition of facial muscle anatomy. This applies to its attachment points and precise three-dimensional positioning within the soft tissue. The aim of this study, as a first step, was to test this hypothesis under optimal conditions and at the highest possible resolution, and eventually establish a new description of the facial musculature.

## 2. Materials and Methods

### 2.1. Human Cadaveric Specimens

For this study, 14 heads of body donors (7 male and 7 female) were obtained in collaboration with the Department of Anatomy I of the University of Jena’s Body Donation Programme for donors aged over 50 (Table 1). The body donors used here were part of a more extensive study aimed at determining the morphological characteristics of the facial muscles in the same individuals using various methods. As this also involved contrast-enhanced imaging and histology, only body donors could be used. The study was approved by the local ethics committee (No. 2020-1661). Furthermore, the guidelines for post-mortem examination of body donors were observed [16].

### 2.2. Standardised Sonography, Computed Tomography and Magnetic Resonance Imaging Workflow

A standardised procedure was applied to all heads during data acquisition, regarding the structures relevant to facial expressions. It was important that the individual steps were coordinated to minimise stress on the tissue. Ultrasound images were acquired immediately after access to the donors’ heads. The ultrasound images were intended to serve as a simple solution link to clinical applications (not presented in this article). Subsequently, the calotte was opened and removed to extract the brain for other research projects. During all intermediate steps, the heads were stored at 4 °C. At the same time, preparations were made for embedding the specimen in a stabilising medium (agar-agar). Stabilisation was necessary as the tissue was unfixed, which could lead to displacement artefacts. The agar-agar (Diagonal, Münster, Germany) was dissolved in water heated to 100 °C at a slightly higher concentration (40 g/3 L to ensure greater stability. In addition, 0.1 g of thymol (Sigma-Aldrich, St. Louis, MO, USA) was added as an antimicrobial agent. After cooling to below 40 °C to prevent tissue damage, the heads were embedded in special plastic buckets (5 L dispenser bucket—Dr. Schumacher, Malsfeld, Germany) containing agar-agar. These plastic buckets were selected for easy handling and to minimise and standardise the surrounding agar volume while maintaining a close fit within the MRI head coil.

The MRI scans were then performed using a 20-channel head coil on a 3T MRI scanner (MAGNETOM Prisma, Siemens Healthineers, Forchheim, Germany). The heads were scanned twice with an isotropic resolution of 640 µm using a 3D T2-weighted turbo spin-echo sequence with variable flip angles (T2 SPACE) (echo time 109 ms, repetition time 1000 ms), once with and once without fat suppression [17]. The total acquisition time in the MRI scanner was between 40 and 60 min.

In addition, a high-resolution computed tomography (CT—Artis Zeego Q system, Siemens Healthineers, Erlangen, Germany) scan of the heads was performed to provide a detailed view of the bony structures. The entire head was scanned within 10 min at a resolution of 488 µm.

The entire processing procedure, including embedding and imaging, took 11 h per head. During the breaks between procedures, strict care was taken to maintain the cold chain. The heads were then removed from the agar-agar.

### 2.3. Data Processing

In summary, the data collection yielded 3D datasets of the bony and soft-tissue structures for all heads. These datasets were subsequently analysed with respect to their modality and individual characteristics. To analyse the distribution of muscle and adipose tissue, the two acquired T2 SPACE sequences were utilised. A dedicated fat-only image volume was generated by digitally subtracting the fat-suppressed T2-weighted images from the standard T2-weighted images, effectively isolating the adipose tissue for further analysis.

Due to the imaging technique used in CT scans, the density of the scanned material could be directly determined. To do this, the CT images were scaled in Hounsfield units (HU), which were converted into material densities. Air had an HU value of −1000, fatty tissue approximately −100, water 0, and bone could range from 500 to 1500. Bone densities are significant because they can indicate load-dependent areas under high forces. Furthermore, it was possible to quantify the general condition of the body donor with respect to bone structure degeneration (see statistics).

### 2.4. Segmentation

The MRI datasets were segmented to identify four different muscle groups: the facial muscles (*Musculi faciei*), masticatory muscles (*Musculi masticatorii*), tongue muscles (*Musculi linguae*) and extraocular muscles (*Musculi externi bulbi oculi*). In what follows, we use the term ‘group’ to refer to these muscles in the head and neck region. We avoided anatomical definitions of facial muscles. When referring to facial muscles in specific regions, we talk, for instance, of ‘*M. orbicularis oris* region’ and not of the ‘*M. orbicularis oris*’. This was carried out by three raters (AR, KJ, HS). The task was to segment the entire facial musculature without paying attention to individual anatomically pre-defined muscles, to avoid a biased or artificial division (e.g., in the *Modiolus anguli oris*). The raters were instructed to segment only the musculature, each applying their own strategy. Preferred strategies included segmenting layer by layer, orienting themselves by muscle-typical intensities, and checking the reconstruction in real time in a 3D view. The free software 3D Slicer (https://www.slicer.org, Version 5, Brigham and Women’s Hospital, Boston, MA, USA) and the commercial software Amira (https://www.thermofisher.com, Version 6, Thermo Fisher Scientific Inc., Waltham, MA, USA) were used individually for this purpose. In 3D Slicer, the slice orientation could also be freely selected. A consensus dataset was generated from the individual segmentations, with a voxel included only if at least two raters agreed [18,19]. For each head, a low-resolution muscle-group map was additionally segmented, with assignments to the facial, masticatory, ocular, and lingual muscles. Furthermore, the bony structures were automatically segmented using thresholds derived from the MRI dataset to the extent possible, enabling co-registration of the CT and MRI datasets.

The segmentation of the bony structures was performed semi-automatically from the CT dataset based on bone HU values, again using the 3D Slicer programme (https://www.slicer.org, Version 5, Brigham and Women’s Hospital, Boston, MA, USA). A surface (3D mesh) was calculated from the segmented data, which served as the basis for local density measurement in the imagexd programme (https://starkrats.de, Version 8, Heiko Stark, Jena, Germany). The local density measurement was calculated at all vertices of the 3D mesh along directions opposite to the normal vectors, and the surface was coloured accordingly. This resulted in a 3D model containing the density gradients, which could be visualised using the free software Blender (https://www.blender.org, Version 3, Blender Foundation, Amsterdam, The Netherlands). All segmented datasets are available at https://doi.org/10.6084/m9.figshare.32324658.

### 2.5. Statistics

**Figure 1:**
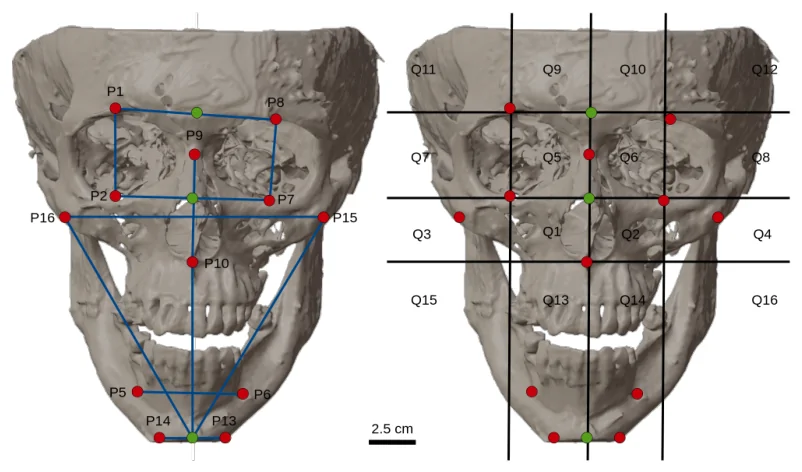
Overview of the markers used (P—red dots: (**left**)) and the 16 quadrants (Q—enclosed areas: (**right**)) for local analysis. The green dots show the mean values between adjacent markers, and the blue lines show the distances used. (P1—right supraorbital foramen; P2—right infraorbital foramen; P5—right mental foramen; P6—left mental foramen; P7—left infraorbital foramen; P8—left supraorbital foramen; P9—upper nasal septum; P10—lower nasal septum; P13—left chin; P14—right chin; P15—left zygomatic arch; P16—right zygomatic arch).

For the muscle measurements, the volume, intensity values, thickness and distance from the bony structures were calculated using the ‘imagexd’ software. To this end, scripts were used to calculate the individual measured values from the respective data sets. The relevant scripts are available via the link (https://github.com/heikostark/3D-FACE-classification, accessed on 12 September 2026). The software enabled various operations (e.g., addition, subtraction, masking) to be performed using image stacks as variables and the results to be statistically analysed. For this purpose, the musculature was divided into facial, masticatory, ocular and tongue muscles and analysed. To classify these groups, the categorisation of the musculature performed by the raters was used, extracted using ‘imagexd’. In order to take into account further local characteristics of the entire facial muscle network beyond anatomical definitions—for example, due to very thin musculature—the facial musculature was additionally subdivided into 16 quadrants using ‘imagexd’ (Figure 1). The quadrants were defined in ‘3D Slicer’ using distinctive landmarks. In addition, further measurements were carried out using the same software to determine the distribution of adipose tissue, and CT scans were used to analyse bone density statistics. The segmented structures were evaluated by gender, age, and height, with median values reported.

Statistical analyses were performed using the freely available statistical software environment R (version 4.2.3). In addition to the base functions, several packages were employed to provide specific analytical and graphical functionalities, including R.matlab, data.table, Rfast, tidyverse, stats, rstatix, car, ggstatsplot, viridis, and openxlsx. Initially, descriptive statistical parameters were calculated for each dataset, including sample size; mean, minimum and maximum values; quantiles; potential outliers; and the distribution of the data. These analyses were performed using the functions ‘get_summary_stats’, ‘identify_outliers’, and ‘shapiro_test’. Subsequently, analysis of variance (ANOVA) was performed to determine whether statistically significant differences existed between the investigated groups using the ‘aov’ function [20]. When significant differences were detected, Tukey’s honestly significant difference (HSD) test was applied as a post hoc analysis to perform all possible pairwise comparisons between groups using ‘TukeyHSD’ [21]. Because homogeneity of variances could not be assumed for all parameters, Levene’s test was additionally performed using the ‘leveneTest’ function. In cases where the assumption of variance homogeneity was violated, pairwise group comparisons were additionally conducted using the Games–Howell post hoc test (’games_howell_test’), which is more robust to unequal variances [22]. Finally, the results were visualized using R. Box-and-whisker plots were generated for all datasets included in the statistical analyses using the ‘ggplot’ function from the ggplot2 package and the ‘ggbetweenstats’ function from the ggstatsplot package. All statistic datasets are available at https://doi.org/10.6084/m9.figshare.33435931.

## 3. Results

### 3.1. Data Quality

**Figure 2:**
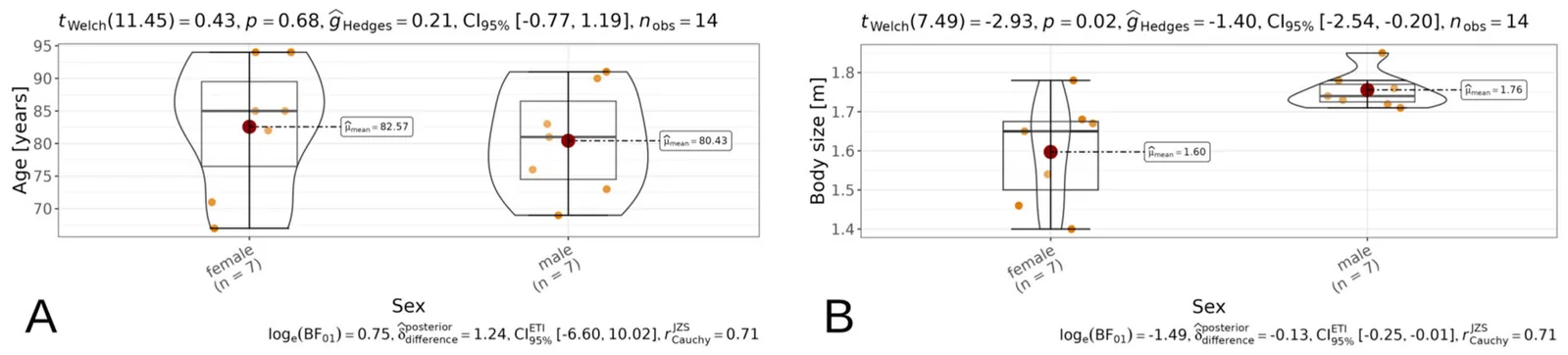
Analysis of variance for age (**A**) and height (**B**) among the women (left side) and men (right side), respectively, using Welch’s one-way ANOVA. Box and violin plots were used to illustrate the data distribution. This was calculated using the ggbetweenstats function from the R package ggstatsplot [23].

Because of the use of body donors, the groups of men (80 ± 8 years) and women (82 ± 10 years) in this study were older than the general population mean (Figure 2). In terms of height (men: 1.76 ± 0.01 m; women: 1.60 ± 0.14 m; Figure 2), they were comparable to the representative German population (male: 1.74 m; female: 1.62 m; https://www.destatis.de) for this age group. A significant gender dimorphism was observed (*p* = 0.02).

Imaging without motion artefacts was made possible by using body donors. Furthermore, MRI contrast was enhanced by the specimens being free of fixative. This helped to prevent shrinkage and a reduction in contrast. However, in some body donors (#5, #9, #10), dental implants, which, in most cases, could not be completely removed, led to oversaturation artefacts in CT scans and extinction artefacts in MRI scans. This primarily affected the teeth and the *M. orbicularis oris* region.

### 3.2. Musculature Volume (MRI Data)

**Figure 3:**
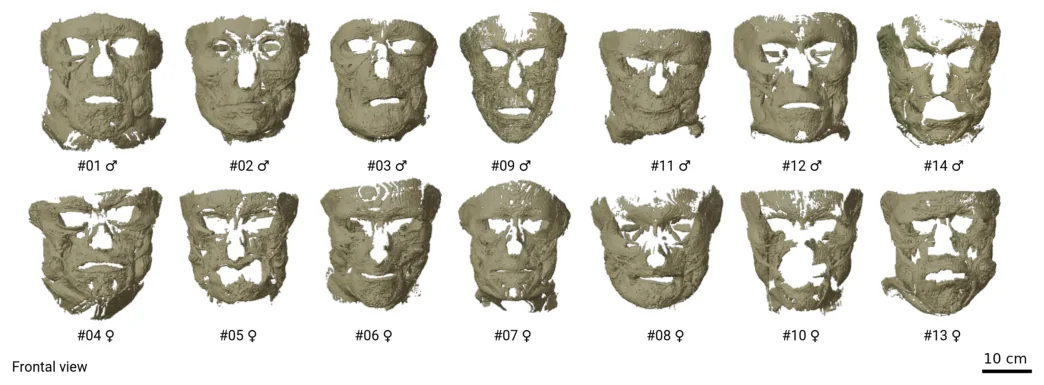
Reconstruction of the entire facial musculature from the 14 body donors (♂: male, ♀: female), based on MRI scans without attempting to delineate anatomically defined facial muscles. The reconstruction is presented as the optimal superimposition of at least two raters.

The muscles were clearly visible on the MRI scan due to their specific signal intensity and fibre structure. Fat suppression provided good delineation from the surrounding tissue. The muscles were segmented without requiring individual anatomically defined muscle identification (Figure 3). The raters were instructed to identify only the facial muscles and clearly distinguishable muscle groups. The muscle groups examined here are the facial, masticatory, extraocular and tongue muscles.

**Figure 4:**
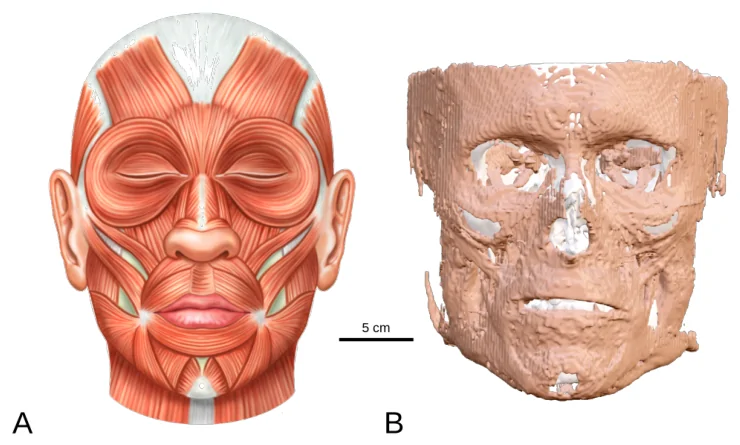
Comparison of the typical frontal view of the facial muscles in anatomy textbooks (**A**) and the muscles segmented, shown as a 3D reconstruction of the MRI dataset from male donor #12 (**B**). The textbook does not clearly reflect the complexity and interdependence of the facial musculature.

The subsequent segmentation showed that the four head and neck muscle groups defined could be easily distinguished from one another automatically using the watershed algorithm. However, with regard to the facial muscle network, it was not possible to distinguish any anatomically defined muscles automatically (Figure 4).

**Figure 5:**
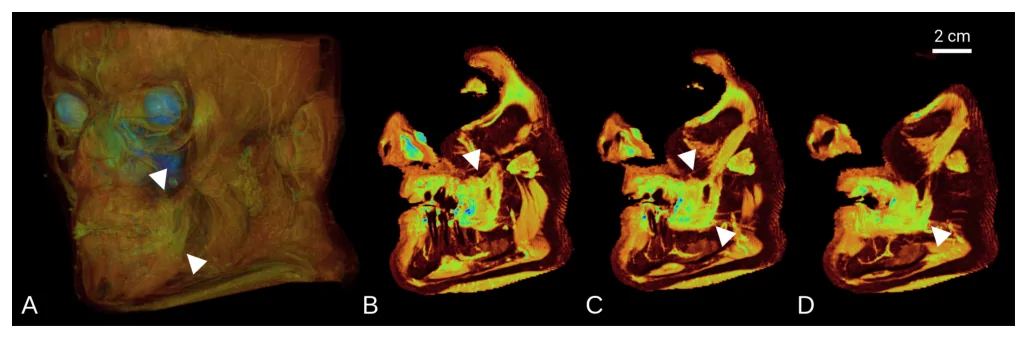
MRI reconstruction of the four defined head and neck muscle groups (facial muscles, masticatory muscles, tongue muscles, and extraocular muscles) of a body donor with fat suppression to illustrate the course of the muscles in *M. zygomaticus* region, which inserts into the *Modiolus anguli oris* (indicated by arrows). To highlight the musculature, the ‘dGEMRIC-3T’ colour palette was used in 3D Slicer. Structures with high water content are shown in shades ranging from brown to yellow to blue. (**A**): General view; (**B**–**D**): sagittal sections at the level of the *Modiolus anguli oris*, from profound to superficial.

The reasons for this were the lack of clearly distinguishable brightness intensities and, consequently, the absence of contrasting structures (e.g., connective tissue or adipose tissue) in the MRI scans. This was particularly evident in the *M. zygomaticus* region, as its insertion into the *Modiolus anguli oris* made it difficult to define its boundaries more precisely (Figure 5).

**Figure 6:**
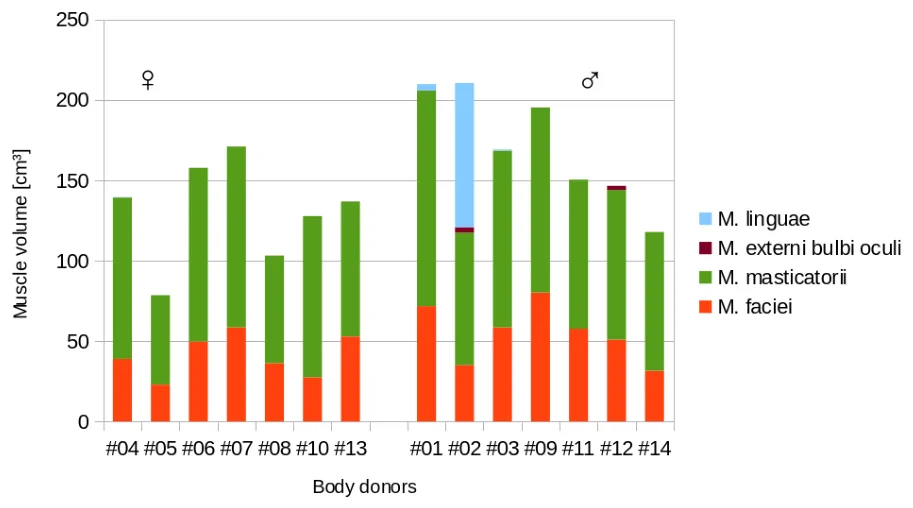
Representation of the volume proportions of the four head and neck muscle groups (facial muscles: red, masticatory muscles: green, tongue muscles: blue, and extraocular muscles: purple) in the 14 body donors. (**Left**): ♀—women; (**right**): ♂—men.

**Figure 7:**
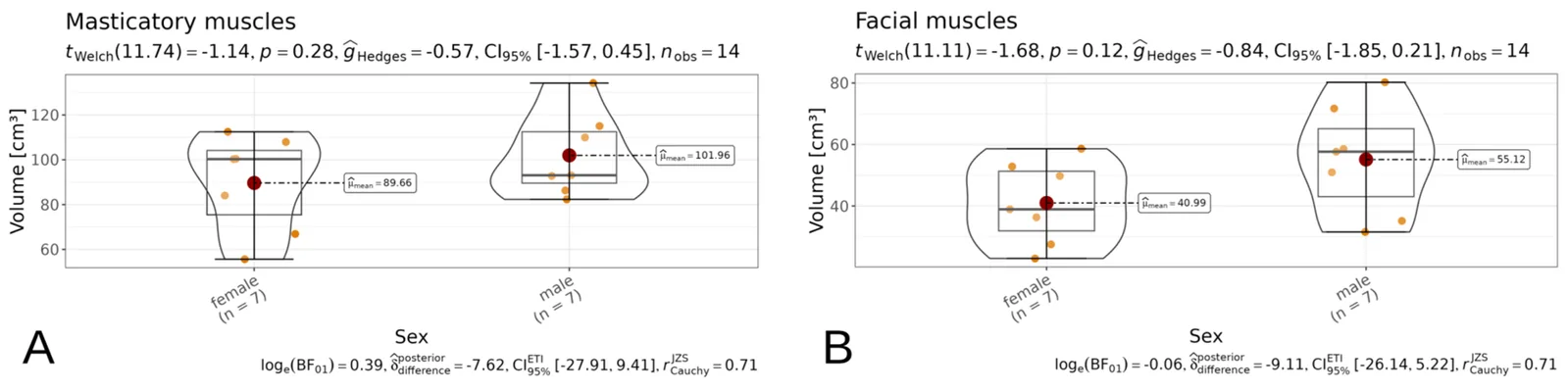
Analysis of variance of muscle volume between the sexes (women: left side; men: right side, respectively) for the masticatory muscles (**A**) and the facial muscles (**B**) using Welch’s one-way ANOVA. Box and violin plots were used to illustrate the data distribution. This was calculated using the ggbetweenstats function from the R package ggstatsplot [23].

By segmenting the musculature, individual muscle groups could be compared with one another (Figure 6 and Figure 7). The facial muscles served as the subject of the study, with the masticatory muscles, which were also fully segmented, used for comparison. On average, the facial muscles were slightly larger in men (men: 55.12 ± 17.78 cm^3^; women: 40.99 ± 13.30 cm^3^); the same applied to the masticatory muscles (men: 101.96 ± 18.56 cm^3^; women: 89.66 ± 21.55 cm^3^). A comparison of the percentages revealed an imbalance between men and women (ratio of facial to masticatory muscles: men: 0.54; women: 0.46). Men, therefore, had a higher proportion of facial muscles than women.

**Figure A2:**
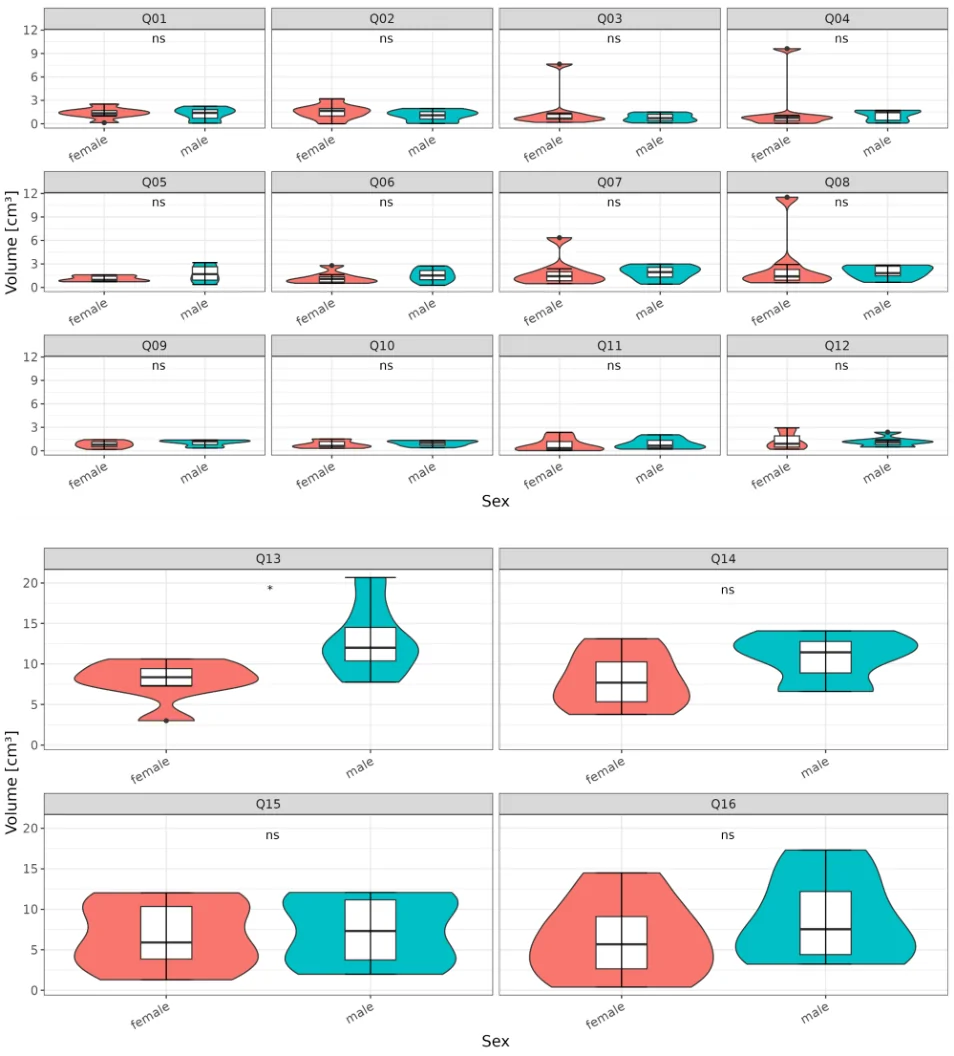
Multiple box and violin plots showing local MRI volume proportions of the facial muscles in the 16 quadrants (*: *p* < 0.05; ns: not significant). (**Top**): For quadrants 1–12—cranial to the nasal septum; (**bottom**): for quadrants 13–16—caudal to the nasal septum; females in red and males in cyan. Statistics: R: stat_compare_means (method = ‘anova’).

A detailed analysis of specific facial regions revealed gender-specific differences in the area around the mouth and chin (Figure A2 below: Q13–Q14). Male body donors exhibited a significantly higher proportion of facial muscles in this area.

### 3.3. Musculature Thickness (MRI Data)

**Figure 8:**
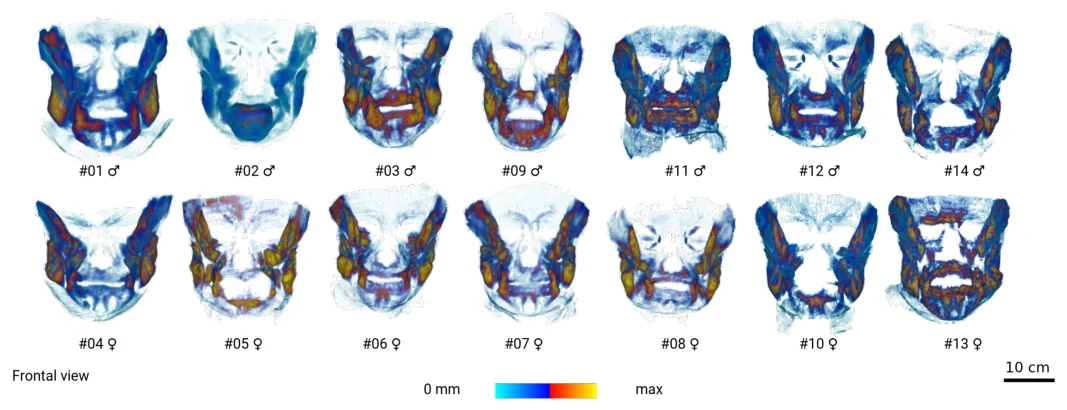
Reconstruction and measurement of the thickness of the facial muscles of the 14 body donors (♂: male, ♀: female) based on MRI scans. The reconstructions show muscle thickness using false-colour coding (blue = thin muscles; red and yellow = thick muscles).

**Figure A3:**
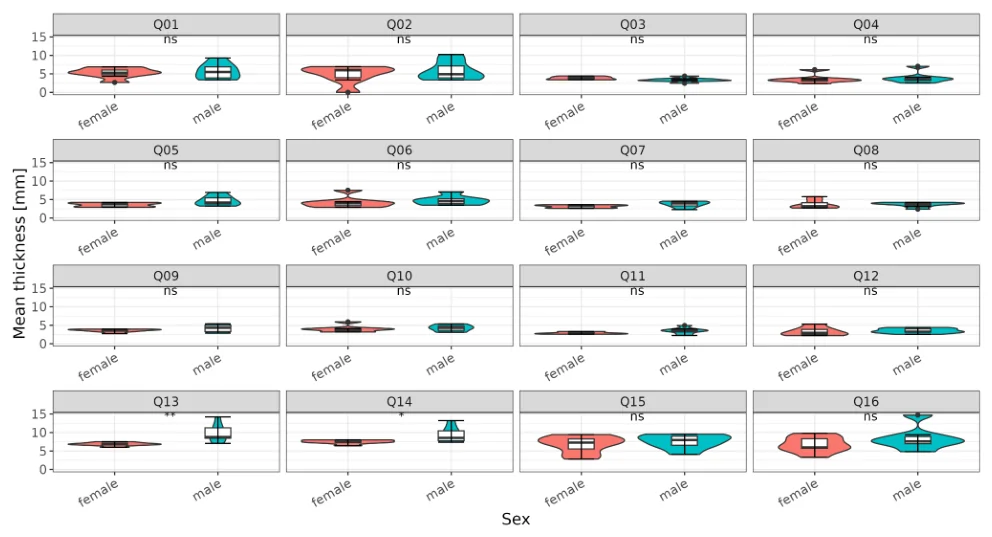
Multiple box and violin plots showing mean MRI local thickness of the facial muscles in the 16 quadrants (**: *p* < 0.01; *: *p* < 0.05; ns: not significant). Females in red and males in cyan. Statistics: R: stat_compare_means (method = ‘anova’).

As expected, muscle thickness was greatest in the *M. temporalis* and *M. masseter* (Figure 8). In the case of the facial muscles, it was more difficult to precisely define the anatomically defined muscles due to the formation of a muscle plate. In this context, we use the term ‘muscle plate’ to refer collectively to all facial muscles that cannot be delineated more precisely and are arranged in a flat configuration. As these muscles originate from the platysma, the only difference here is their internal organisation compared with the platysma as a muscle plate. However, the *Modiolus anguli oris* could be identified as a connecting centre between various muscles, as well as in the region of the *M. orbicularis oris*, where increased muscle thickness was observed. In contrast, the flat and thin muscles were found in the region of the *M. frontalis*, *M. orbicularis oculi* and the *Platysma* (Figure A3: Q9–Q12). Thickness measurements revealed the same significant gender differences in the facial regions as in the oral area (Figure A3: Q13–Q14).

### 3.4. Fat Tissue (MRI Data)

**Figure A5:**
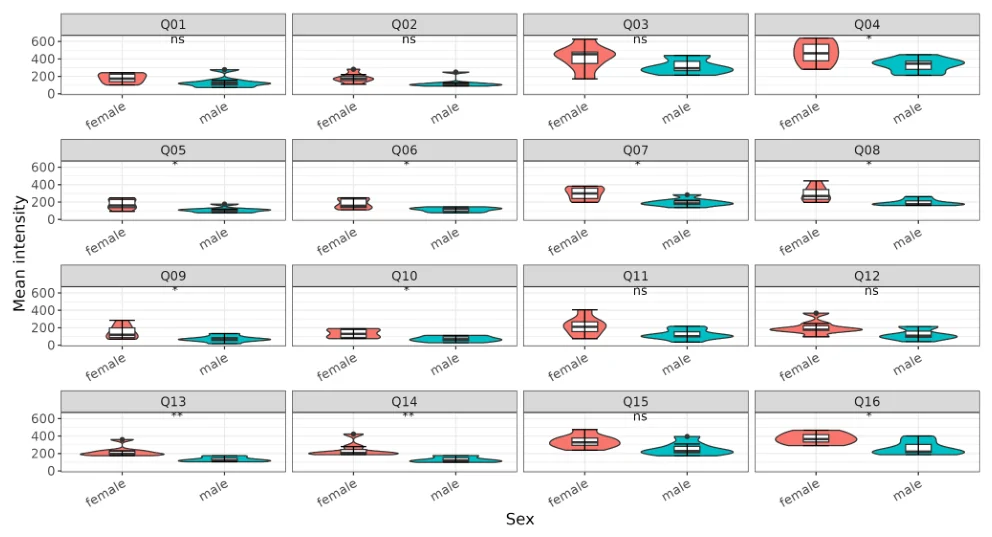
Multiple box and violin plots showing local MRI intensity values of fatty tissue in the 16 quadrants (**: *p* < 0.01; *: *p* < 0.05; ns: not significant). (**Top**): For quadrants 1–12—cranial to the nasal septum; (**bottom**): for quadrants 13–16—caudal to the nasal septum; females in red and males in cyan. Statistics: R: stat_compare_means (method = ‘anova’).

**Figure A6:**
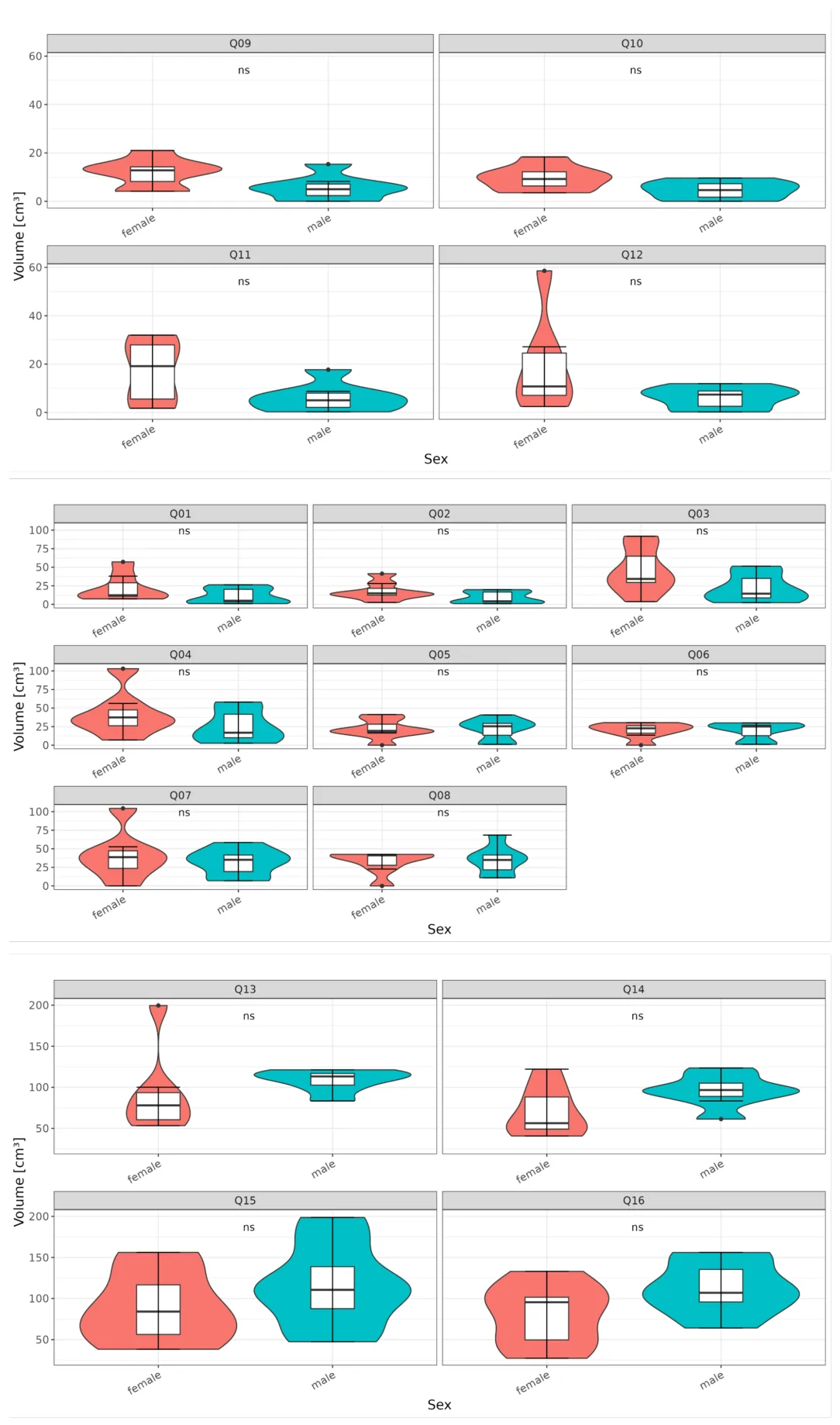
Multiple box and violin plots showing local MRI volume proportions of fatty tissue in the 16 quadrants (ns: not significant). (**Top**): For quadrants 9–12—cranial to the orbit; (**middle**): for quadrants 1–8—orbit + cranial to the nasal septum; (**bottom**): for quadrants 13–16—caudal to the nasal septum; females in red and males in cyan. Statistics: R: stat_compare_means (method = ‘anova’).

Using specialised MRI imaging with fat saturation, the fatty tissue could be segmented solely by subtracting it from a non-fat-saturated image. This made it possible to analyse the data directly without the need for multiple raters. With regard to volume, a potential division into three zones was observed (cranial to the orbit; orbit + nose; caudal to the nasal septum). The smallest volume was found cranial to the orbit in each quadrant (5.60 cm^3^–19.30 cm^3^), and the largest volume caudal to the nasal septum (70.56 cm^3^–115.63 cm^3^). In all three zones, no significant sex dimorphism was observed with regard to gender (Figure A5 and Figure A6). Nevertheless, a trend towards higher fat content was observed in female body donors cranial to the orbit and in male donors caudal to the nasal septum.

**Figure 9:**
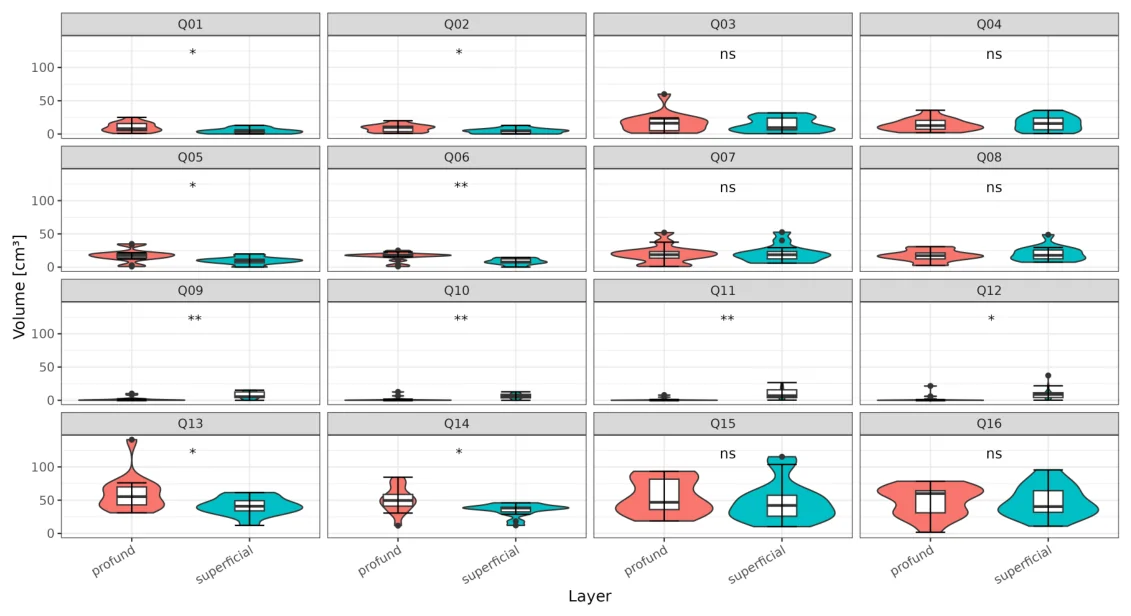
Multiple box and violin plots of the volume proportions of fatty tissue, distinguished by facial muscles, with regard to profound (red) deep and superficial (cyan) fatty tissue (**: *p* < 0.01; *: *p* < 0.05; ns: not significant). (Quadrant division as per Figure 1.) Statistics: R: stat_compare_means (method = ‘anova’).

**Figure A7:**
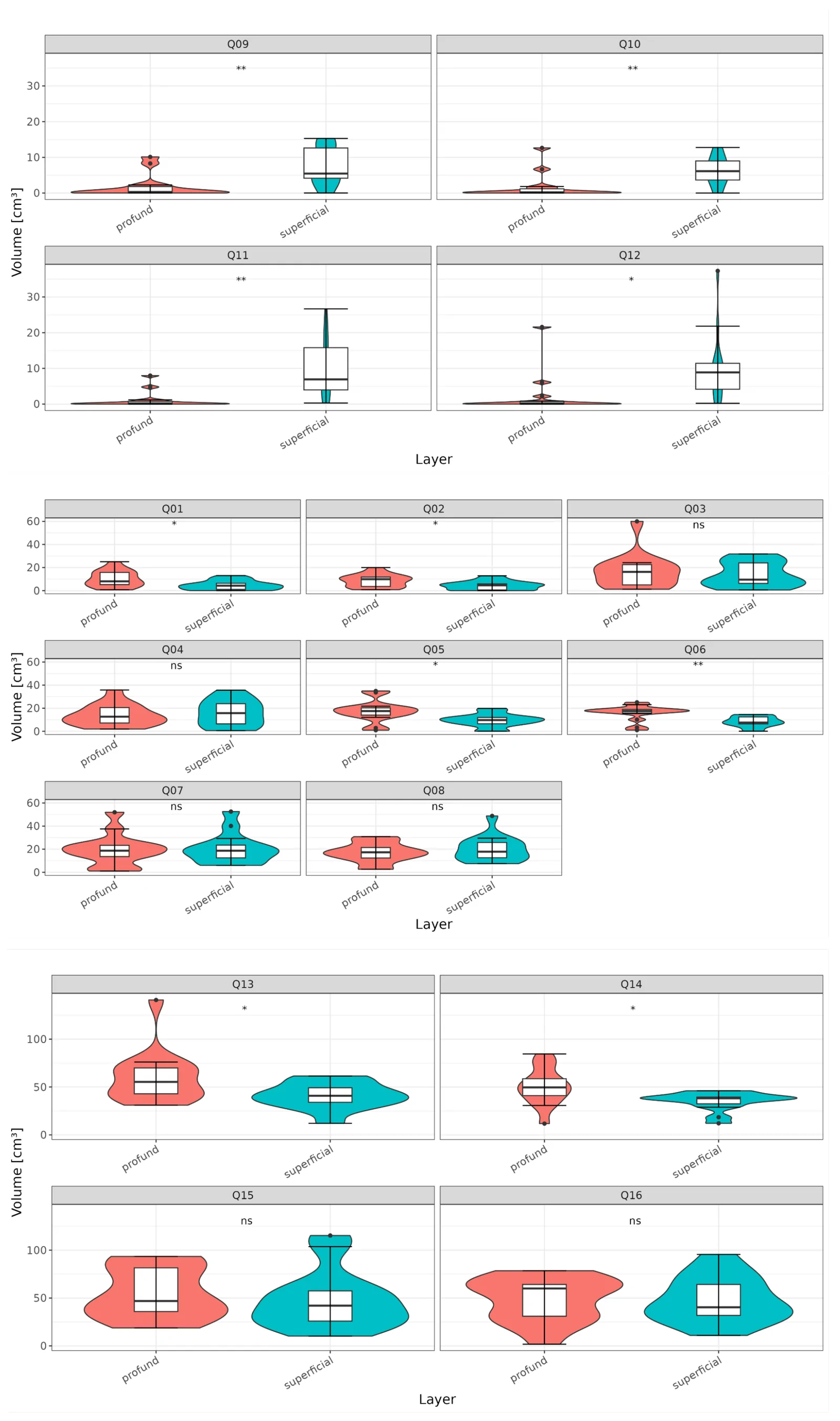
Multiple box and violin plots showing local MRI volume proportions of fatty tissue in the 16 quadrants, distinguished into profound and superficial fatty tissue (**: *p* < 0.01; *: *p* < 0.05; ns: not significant). (**Top**): For quadrants 9–12—cranial to the orbit; (**middle**): for quadrants 1–8—orbit + cranial to the nasal septum; (**bottom**): for quadrants 13–16—caudal to the nasal septum; females in red and males in cyan. Statistics: R: stat_compare_means (method = ‘anova’).

As the muscle tissue was embedded in adipose tissue, a distinction was made here between profound and superficial fat (Figure 9 and Figure A7). Significant differences were observed in the forehead region (Q9–Q12) and the cranial nasal region (Q1–Q2 & Q5–Q6); however, these should be interpreted as artefacts due to the difficulty of segmenting the musculature and the low proportion of soft tissue. In contrast, a significantly higher fat content was observed in the mouth and chin region (Q13–Q14).

### 3.5. Bones (CT-Data)

**Figure 10:**
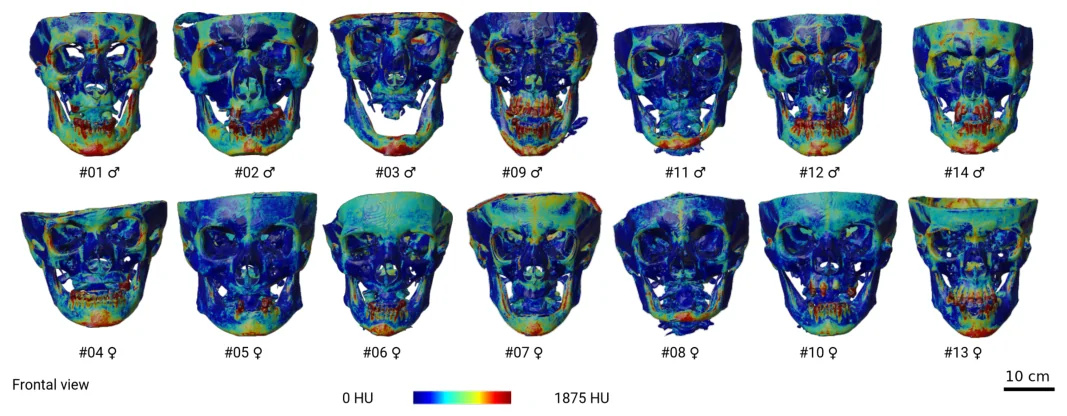
Reconstruction of the bony structures of the skulls of the 14 body donors (♂: male, ♀: female) based on CT scans. The structures are coloured according to local bone density using the Hounsfield scale (HU)—low density (blue) or high density (red).

**Figure A8:**
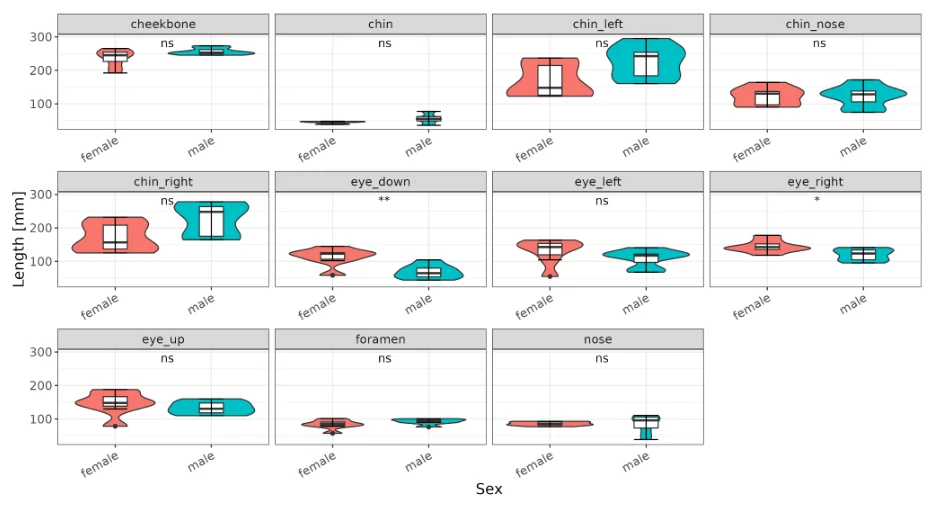
Multiple box and violin plots showing a comparison of selected distances between the 16 markers by gender (**: *p* < 0.01; *: *p* < 0.05; ns: not significant). Cheekbone: P15–P16; Chin: P13–P14; Chin_left: ((P13 + P14)/2)—P15; Chin_nose: ((P13 + P14)/2)—P10; Chin_right: ((P13 + P14)/2)—P16; Eye_down: P2–P7; Eye_left: P7–P8; Eye_right: P1–P2; Eye_up: P1–P8; Foramen: P5–P6; Nose: P9–P10; females in red and males in cyan. Statistics: R: stat_compare_means (method = ‘anova’).

**Figure A9:**
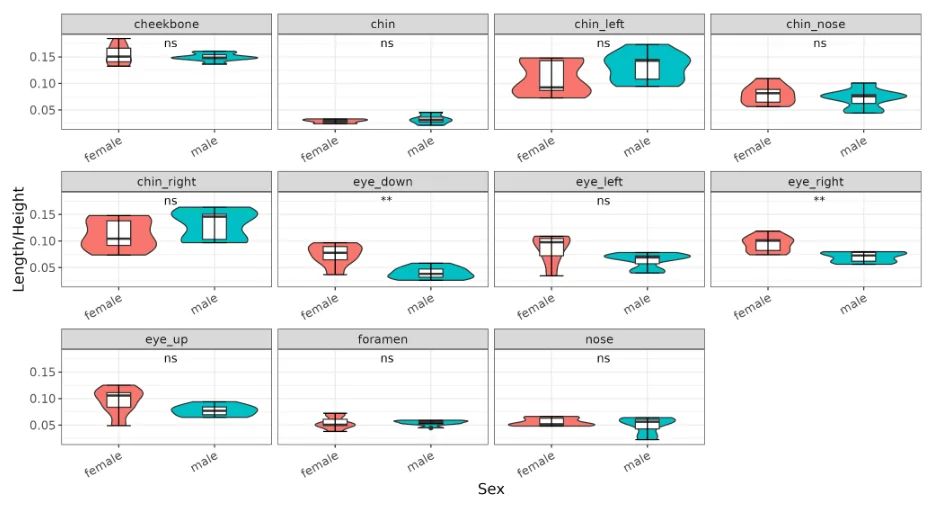
Multiple box and violin plots showing a comparison of selected body measurements of normalised distances between the 16 markers by gender (**: *p* < 0.01; ns: not significant). Cheekbone: P15–P16; Chin: P13–P14; Chin_left: ((P13 + P14)/2)—P15; Chin_nose: ((P13 + P14)/2)—P10; Chin_right: ((P13 + P14)/2)—P16; Eye_down: P2–P7; Eye_left: P7–P8; Eye_right: P1–P2; Eye_up: P1–P8; Foramen: P5–P6; Nose: P9–P10; females in red and males in cyan. Statistics: R: stat_compare_means (method = ‘anova’).

The skull bones could be reconstructed directly from the CT data (Figure 10). Because this imaging technique measures tissue density, bone tissue could be automatically segmented based on its high density. Subsequently, only artefacts (e.g., dental implants) needed to be removed. Based on this reconstruction, distinctive landmarks were defined for morphometric analyses (Figure 1). This enabled comparison of distances between morphological structures (Figure A8 and Figure A9).

This comparison revealed a significant difference in gender-specific inter-eye distance in both the absolute and normalised data. Women, therefore, generally had a greater distance between their eyes. Regarding the orbit, women tended to have larger eye sockets, with the right eye socket larger in absolute terms and the left eye socket significantly larger in normalised terms.

**Figure 11:**
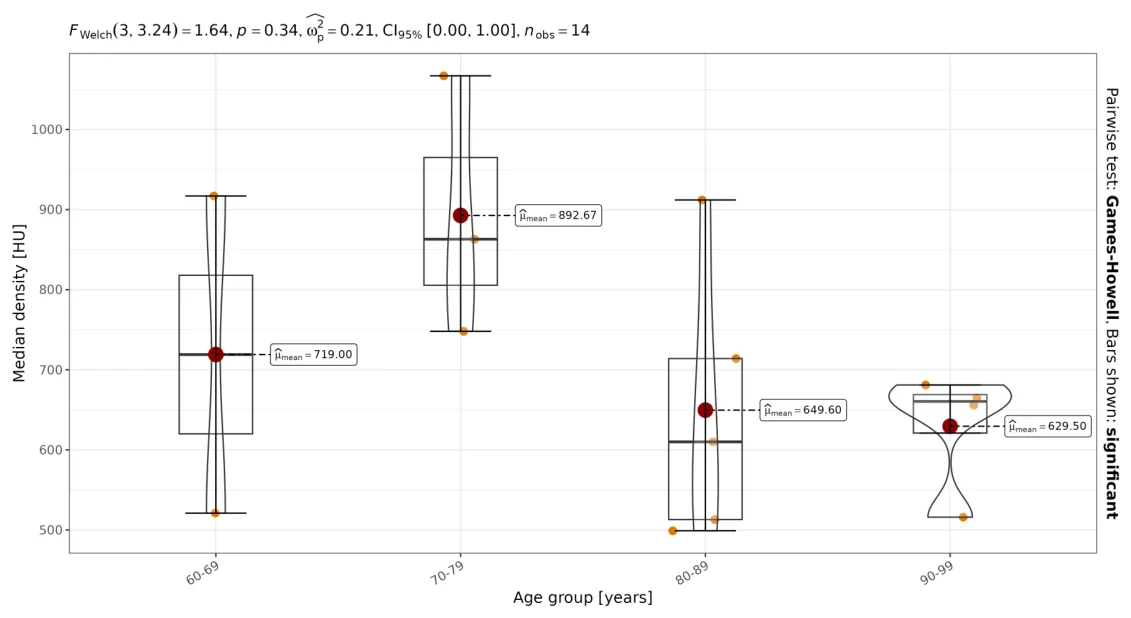
Analysis of variance of CT skull bone density in relation to the age of body donors (age groups from left to right: 60–79, 70–79, 80–89, 90–99 years) using Welch’s one-way ANOVA. Box and violin plots were used to illustrate the distribution of the data. This was calculated using the ggbetweenstats function of the R package ggstatsplot [23].

**Figure A10:**
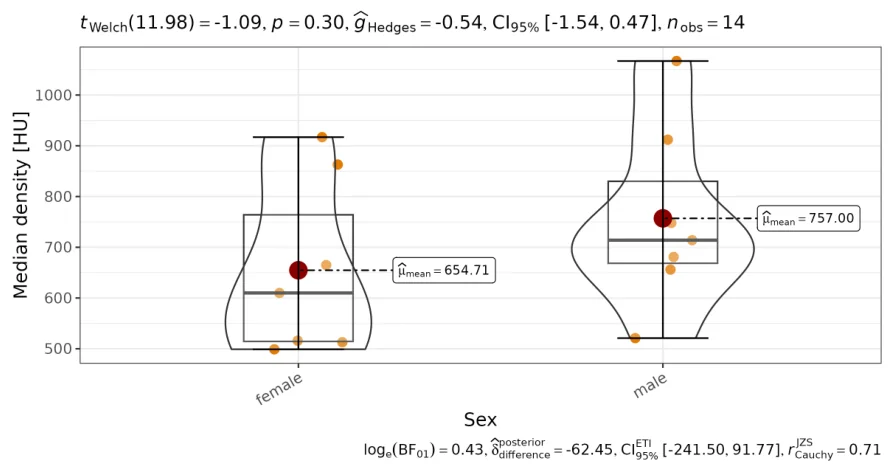
Box and violin plots showing computed tomography (CT) bone density (median) in a comparison between women (**left side**) and men (**right side**), as measured on the Hounsfield scale [HU]. Statistics: R: ggbetweenstats.

**Figure A11:**
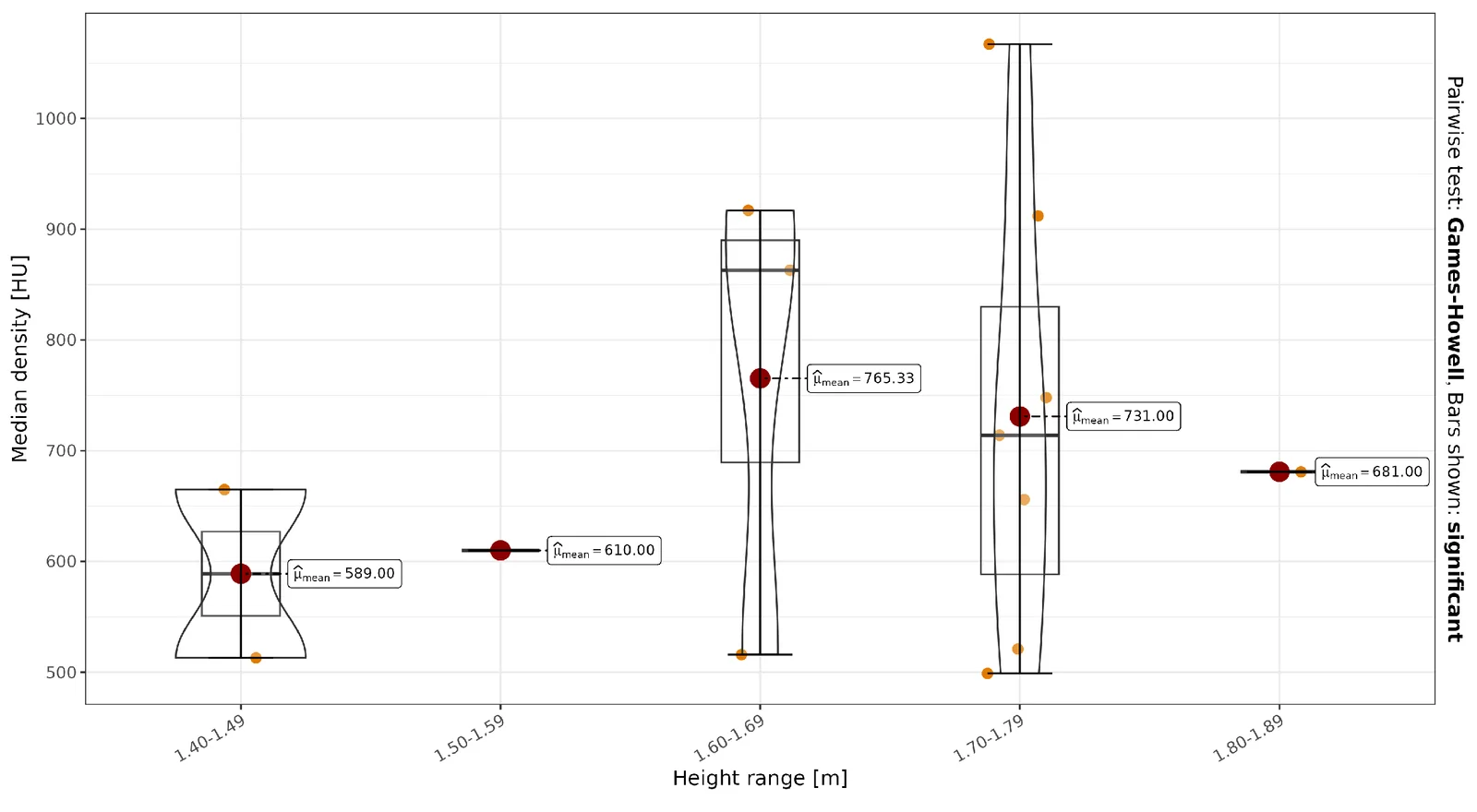
Box and violin plots showing computed tomography (CT) bone density (median) relative to body height, measured on the Hounsfield scale [HU]. Statistics: R: ggbetweenstats.

Based on CT scan density data, differences in bone density were identified by age, sex, and height (Figure 11, Figure A10 and Figure A11). The results showed that younger body donors had higher bone density than those over 80 years of age. There was no significant difference between the sexes, and taller body donors had higher bone density than shorter ones.

### 3.6. Bones vs. Musculature Distance

**Figure 12:**
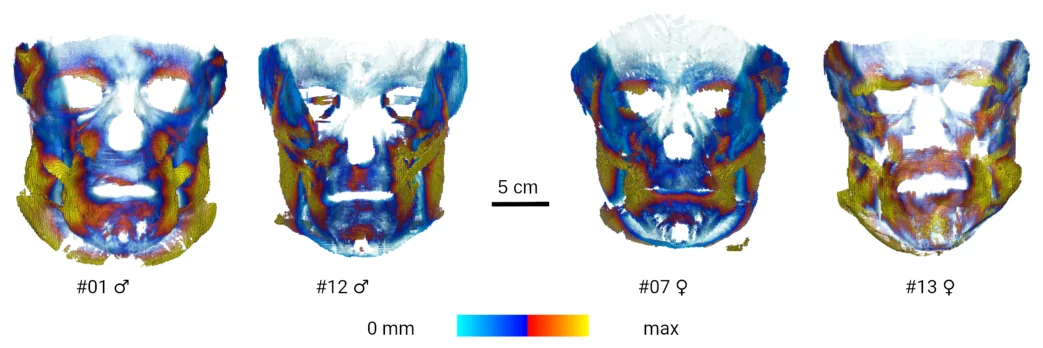
Reconstruction and measurement of the distance between the facial muscles and the underlying bone in four body donors (♂: male, ♀: female), based on CT and MRI scans. The reconstructions depict the distances using false-colour coding (blue = close; red–yellow = far).

To characterise the facial muscles in terms of their attachment to the bone, a measurement of the distance between the structures was carried out. This made it possible to visualise potential connectivity to the bone via connective tissue, intervening fatty tissue, or glands. This connectivity is important because without it, force must be transmitted through connections between muscles. The areas with the thinnest muscles were also the areas where the muscles lay closest to the bone (Figure 12).

## 4. Discussion

The aim of this study was to re-evaluate the facial muscles in relation to the traditional definition of facial muscle anatomy and relevant parameters in clinical practice. This focused in particular on their position within the surrounding tissue and their attachment to the face, independent of anatomically defined, separated facial muscles. In contrast to earlier studies, a higher resolution enabled detailed segmentation of the entire facial muscle network and other head and neck muscle groups [24,25].

### 4.1. Body Donors

The clinical imaging used in this study revealed structures and their locations. This allowed bony structures to be imaged at a resolution of 488 µm and soft tissue at 640 µm in their natural state. The resolution made it possible to visualize the facial muscle structures, even though the conventional, anatomically defined facial muscles could not be distinguished from one another. Furthermore, fixation artefacts [26], which mainly manifest as shrinkage or contrast reduction, were avoided. Measurements on body donors also yielded high-quality images, with no motion artefacts. Despite being very old, the body donors were representative of their age group.

### 4.2. Muscle Sheet

An important finding from the data collection was that, using MRI imaging, the facial muscles could not be divided into individual muscles as depicted in the standard anatomical atlas, particularly in areas where several muscles are interconnected. This can be attributed to the MRI resolution used, although four different head and neck muscle groups, the masticatory, extraocular, tongue and facial muscles, could be clearly distinguished from one another (including automatically). However, this also occurs in other muscles, such as the deep back muscles [13,14]. Consequently, it was also not possible to clarify whether the facial musculature constitutes a single continuous muscle or consists of many individual muscles connected by connective tissue. Rather, the methods used only allow for the identification of a single muscle plate. This was embedded in a layer of fat and was not attached to the bone. The extent to which underlying or overlying adipose tissue influences facial expressions warrants clarification in a subsequent study [27]. The facial bones also formed the basis for the movement of the facial muscles within the fatty tissue. Thus, all three components are essential for describing facial expressions. In a direct comparison of volume proportions, men had a higher proportion of muscle, a pattern also observed in skeletal muscle [28]. Interestingly, gender differences in terms of muscle and fat tissue are greatest in the chin area.

### 4.3. Specific Facial Muscles

The muscles most difficult to segment are those in the region of the *M. orbicularis oculi*, the *M. frontalis* and the *Platysma*. This was clearly due to the resolution limitations of 3T MRI. All three are characterised by very thin muscle layers and cause planar displacement when contracted [29,30]. The area of the *M. orbicularis oculi* was notable for its very thin, flat, circular structure, which is responsible for closing the eyes [31,32,33]. All other muscles are characterised by larger volumes. In particular, the region of the *M. orbicularis oris* is notable for its size and its function in closing the mouth. However, this classification can only be concluded from its function, as the segmentation of individual muscles was subject to classification uncertainties. Only the region of the *M. zygomaticus* (ZMa: major; ZMi: minor) can be described as truly easy to segment. It is therefore not surprising that it has most frequently been the subject of research studies [15,34]. However, it exhibits high variability in position and morphology [34]. Up to five main morphological variants have been described for this. These are, in order of frequency: single-bellied, bifid ZMa, with atypical insertion, accessory ZMi bands and multibellied ZMa. However, based on the MRI data, a precise delineation of its insertion into the *Modiolus anguli oris* was questionable.

### 4.4. Caveats

Using the methods applied here, it was difficult or impossible to distinguish the facial muscles into the traditionally identified and named muscles. However, this finding is significant, as only the imaging techniques used here are available to patients and under more difficult circumstances. Consequently, it is not possible to deduce the directions of force, which are determined by the fascicles, directly from the geometries. Furthermore, there were many areas where the musculature (e.g., in the region of the *M. orbicularis oculi*) was so thin that it fell below the resolution of the imaging (<640 µm). The fascicles and the resolution limit cannot be addressed here. Dental implants presented a particular challenge and were to be removed where possible. They significantly affected the results of the MRI and CT scans.

## 5. Conclusions

In summary, clinical imaging is suitable for visualising the entire facial muscle network. This is important for modelling and subsequent simulation, enabling verification of functional relationships in living subjects. However, the required resolution was not achieved to verify traditional muscular topography or examine muscle connections. Further studies with higher resolution are therefore required.

## Figures and Tables

**Table 1:** Overview of the body donors used, by gender, age, height, cause of death (according to the death certificate) and International Statistical Classification of Diseases and Related Health Problems (ICD-10-GM). (#—number; ♂—male; ♀—female).

N	Sex	Age	Height	Cause of Death	ICD-10-GM
		[Years]	[m]		
#1	♂	69	1.76	Gastrointestinal hemorrhage, generalized vascular sclerosis	K92.2; I70.9
#2	♂	91	1.73	Acute respiratory failure, delirium, subcortical arteriosclerotic encephalopathy, hairy cell leukemia, paroxysmal atrial fibrillation	J96.0; F05; I67.3
#3	♂	90	1.85	Alzheimer’s disease, coronary heart disease, type II diabetes mellitus	I67.2; C91.4; I48.0
#4	♀	71	1.68	Acute and chronic respiratory failure, pulmonary hypertension, nosocomial pneumonia	J96.0; J96.1; I27.2; J18.9
#5	♀	94	1.46	Heart failure, chronic obstructive pulmonary disease, dementia	I50.9; J44.9; F03.9
#6	♀	85	1.54	Acute respiratory failure, central pulmonary embolism (right), infarct pneumonia (right), dementia, type II diabetes mellitus	J96.0; I26.9; J18.9; F03.9; E11.9
#7	♀	82	1.78	Hemorrhagic shock, hematemesis, gastrointestinal bleeding	R57.1; K92.0; K92.2
#8	♀	85	1.40	Progressive hypertensive encephalopathy, arterial essential hypertension, dementia, plasmacytoma	I67.4; I10; F03.9; C90.3
#9	♂	76	1.74	Pancreatic carcinoma, liver metastases	C25.9; C78.7
#10	♀	94	1.65	Acute renal failure, dehydration, dementia, following right breast cancer, arterial hypertension, cardiac insufficiency	N17.9; E86; F03.9; Z85.3; I10; I50.9
#11	♂	83	1.71	Septic multiple organ failure, aspiration pneumonia, strangulated ileus, atrial fibrillation	R65.1; J69.0; K56.2; I48.9
#12	♀	73	1.78	Global end-stage heart failure, bronchopulmonary infection, delirium associated with encephalopathy, aortic valve replacement, cardiorenal syndrome	I50.9; J18.9; F05; G93.4; Z95.2;I50.9
#13	♀	67	1.67	Heart attack, hypertension, diabetes mellitus	I21.9; I10.9; E14.9
#14	♂	81	1.72	Peritoneal carcinomatosis, sigma carcinoma, hypertension, chronic kidney failure	C78.6; C18.7; I10.9; N18.4

**Figure A1:**
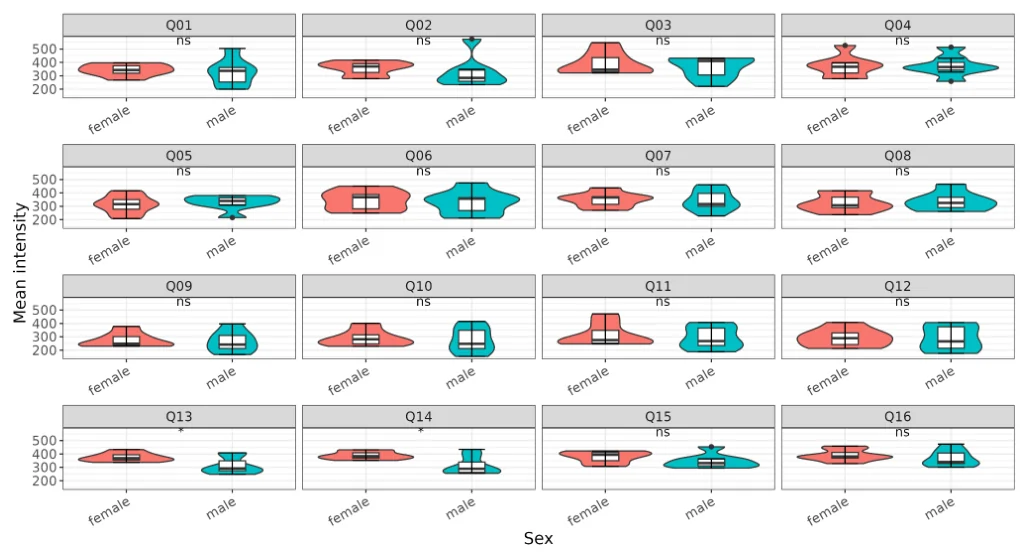
Multiple box and violin plots showing local intensity values in magnetic resonance imaging (MRI) of the facial muscles across the 16 quadrants (*: *p* < 0.05; ns: not significant). (**Top**): For quadrants 1–12—cranial, the nasal septum; (**bottom**): for quadrants 13–16—caudal, the nasal septum; females in red and males in cyan. Statistics: R: stat_compare_means (method = ‘anova’).

**Figure A4:**
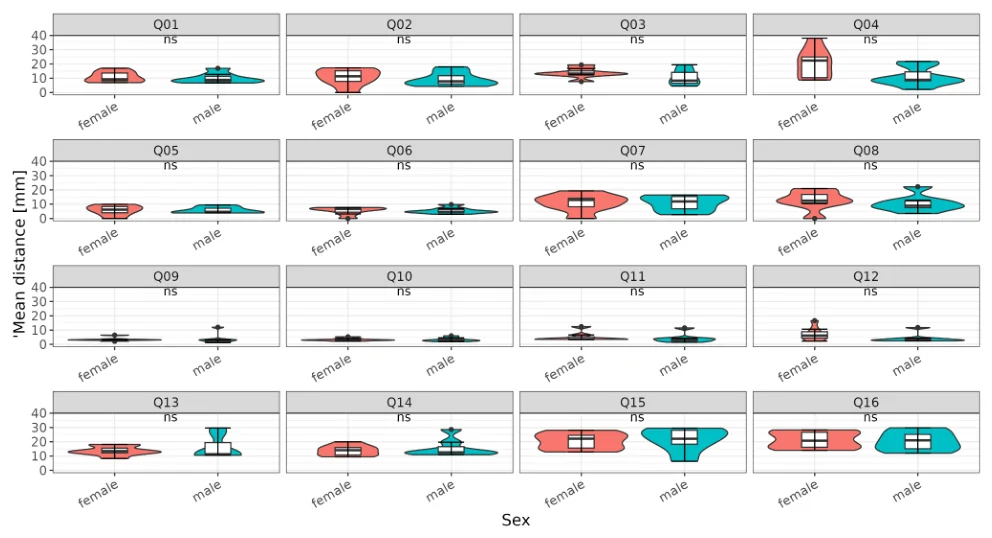
Multiple box and violin plots showing mean MRI local distances between the facial muscles and the underlying bones in the 16 quadrants (ns: not significant). Females in red and males in cyan. Statistics: R: stat_compare_means (method = ‘anova’).

## Data Availability

The data supporting the findings of this study are available from the corresponding author and at https://doi.org/10.6084/m9.figshare.32324658.

## Abbreviations

The following abbreviations are used in this manuscript:

CT	computed tomography
MRI	magnetic resonance imaging
3T MRI	3 Tesla magnetic resonance imaging
HU	Hounsfield units
ZMa	*M. zygomaticus major*
ZMi	*M. zygomaticus minor*
